# Oxidative Stress Induces Telomere Dysfunction and Shortening in Human Oocytes of Advanced Age Donors

**DOI:** 10.3390/cells10081866

**Published:** 2021-07-23

**Authors:** Paweł Kordowitzki

**Affiliations:** Institute of Animal Reproduction and Food Research of Polish Academy of Sciences, Tuwima Street 10, 10-243 Olsztyn, Poland; p.kordowitzki@pan.olsztyn.pl

**Keywords:** oxidative stress, ROS, telomeres, chromosome instability, oocytes, aging

## Abstract

Research from the past decades provided strong evidence that in humans the pool of oocytes starts to decline already before the birth of a female individual, and from menarche to menopause the oocyte is exposed to different environmental stimuli. Since more and more women of the 21st century in developed countries wish to postpone the first pregnancy to their thirties, higher rates of miscarriage and chromosomal non-disjunction might occur. In oocytes of advanced maternal age, meaning above 35 years of age, characteristics such as chromosomal instabilities/abnormalities, spindle defects, decreased mitochondrial function and telomere shortening become more prevalent than in younger counterparts. Telomere attrition belongs to the so-called “hallmarks of aging” which are also relevant for the female germ-line cells. In oocytes, telomeres shorten with advancing maternal age due to the effects of reactive oxygen species and not upon replicative senescence, similar to how it is common in dividing cells.

## 1. Introduction

Telomere attrition and dysfunction are recognized as a hallmark of aging [1] since they are well-established phenomena contributing to organismal aging and telomere homeostasis is a contributor to other processes of aging, too. However, the implication of telomere biology in oocyte aging and women’s fertility is barely starting to be unveiled. Due to changing demographics in the last years, meaning the shift of women’s age having their first baby, the correlation between the accumulation of short telomeres, reactive oxygen species (ROS) and ovarian senescence has emerged [2,3]. Therefore, researchers provided evidence that telomere dysfunction and the decreasing activity of the telomerase in ovarian cell types and oocytes is a common property of women’s sub-and infertility with regards to advanced maternal age [4]. Therefore, in this commentary piece, the most recent knowledge about the correlation between ROS, telomere dysfunction and chromosome instability in oocytes of women will be provided since age-related fertility decline could represent an interesting model to study fundamental phenomena contributing to organismal aging in humans.

## 2. Telomeres and the Aging Oocyte

**Figure 1:**
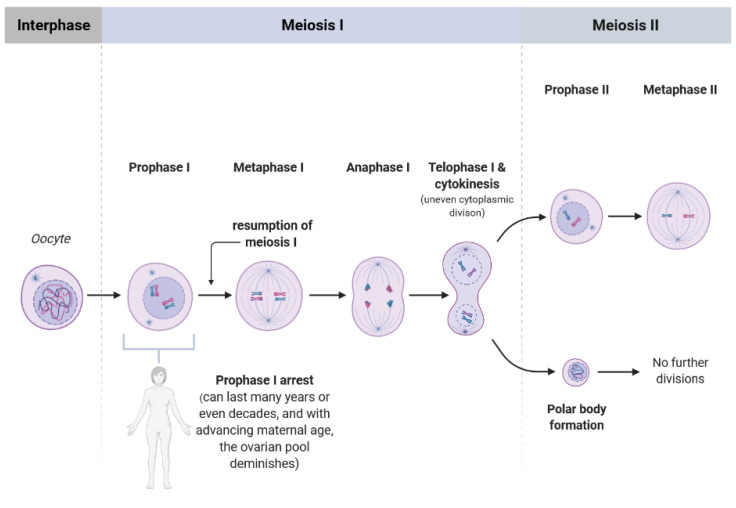
Scheme showing the single phases of meiosis I and II. Every girl (as most of the mammalian species) is born with a defined set of primordial follicles containing the oocytes, however, already at the onset of puberty this ovarian pool has diminished substantially. The oocytes are arrested in prophase I, and from menarche to menopause some of the oocytes are selected for further development when resumption of meiosis occurs. Thereby, the “waiting time” of an oocyte to be fertilized after reaching metaphase II could last many years, nowadays even up to 35 years or more, depending on women’s wish to become pregnant. In consequence, the chance and number of telomeric DNA damage and telomere dysfunction increase with advancing maternal age of the oocyte.

**Figure 2:**
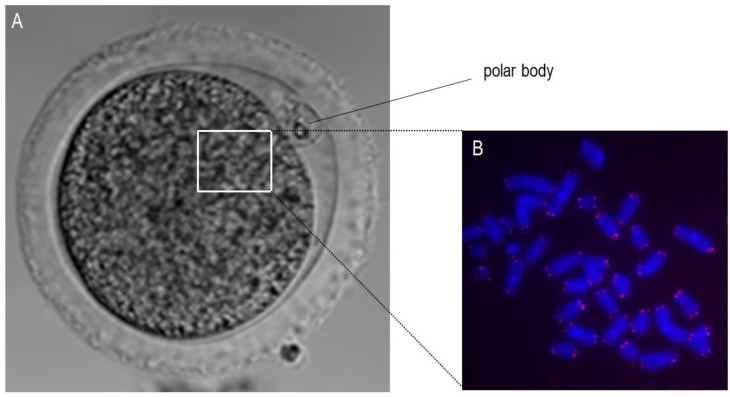
Microscopic visualization of the metaphase II oocyte and chromosomes. (**A**): This panel shows an exemplary bright-field microscopic picture of a bovine oocyte in metaphase II with the visible extruded polar body. The white square shows the approximate localization of the chromosomes. (**B**): This panel shows the stained chromosomes (blue) and telomeres (red) using the Q-FISH method.

Telomeres attracted researchers’ attention since they are crucial for chromosomal stability, meaning for the organization of mammalian genomes into linear chromosomes, and age-related telomere shortening can serve as a biological clock of a cell or organism [5]. The regulation of telomere length in mammals requires the defined interplay between telomeric protein complexes (shelterin) and the ribonucleoprotein telomerase which is responsible for telomere elongation by adding consecutive 5′-TTAGGG-3′ repeats [6]. More precisely, there is a wide range of tasks for which telomeric proteins are responsible, among others they modify the telomere’s architecture, and orchestrate the process during which the newly generated telomeric single-stranded DNA tail is changed into double-stranded DNA [7]. Consequentially, it appears obvious that a reduced function of telomeres in oocytes, meaning the dysfunctional telomere maintenance upon the exposure to ROS leads to telomere shortening. Recent studies postulate that there is a dynamic nature of the telomere architecture and new insights described telomerase-related interactions [7]. Due to the fact that a girl is born with a defined pool of ovarian follicles which gradually diminishes throughout life, oocytes experience chronic exposure to ROS (Figure 1). Moreover, the longer an oocyte is arrested in prophase I, “waiting” to be selected for ovulation (in nowadays demographic situation even more than three decades), the longer is the exposure time of ROS towards oocytes. Following the telomere theory of reproductive aging [8,9] the age-related oocyte dysfunction in women is caused by the progressive telomere shortening since telomeres in oocytes begin to shorten already during oogenesis in the fetal phase [9]. Moreover, as recently reviewed by van der Reest et al. [3], with advancing maternal age an accumulation of mitochondrial DNA mutations occurs leading to respiratory chain deficiency and increased level of ROS. Further, functional telomeres are required for the metaphase chromosome alignment and integrity of meiotic spindles [10]. The physiological segregation of chromosomes during meiosis (Figure 1) after reaching puberty is related the chiasmata formed during fetal life. Importantly though, chiasmata deliver counterattraction alongside spindle-pulling forces to enable kinetochores to secure connections to spindle fibers [11]. Due to Telomere function is essential for meiosis The length of telomeres in metaphase II oocytes (during which the metaphase-plate of chromosomes is established and the first polar body is extruded) can be estimated via the labeling of telomeric repeats with a probe using the Q-FISH method (Figure 2). In human newborns, the telomere length is around 10–15 kb [12]. Therefore, there is a gross need for a better understanding in science and reproductive medicine, how to prevent oocyte aging by minimizing ROS or upon the supplementation with antioxidants.

## 3. Oxidative Stress Impact on Telomeres in Oocytes

**Figure 3:**
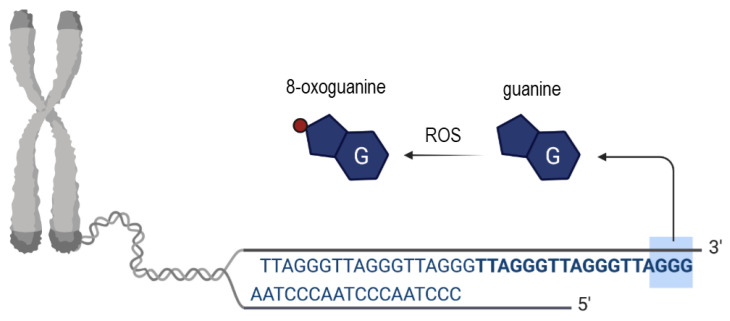
Scheme showing the origin and consequences of oxidative telomeric DNA base damage. Upon ROS the in situ oxidation of a guanine DNA base takes place leading to the generation of 8-oxoguanine (place of oxidation depicted as red dot).

Interestingly, although it has been generally accepted that several environmental causes are responsible for telomere dysfunction and shortening, oxidative stress (OS) appears to be the most cited mechanistic reason for the before-mentioned phenomenon. Mitochondrial dysfunction accounts for one possible explanation for the generation of ROS due to a lack of the antioxidant defense mechanism in the oocyte [13]. More precisely, ROS are generated as a by-product of several reactions in which enzymes contribute, similar to, for instance, the NADPH oxidase enzymes [14,15]. Noteworthy, the neutralization of ROS is important not only in the oocyte since an imbalance provoked by excess ROS production will damage cellular DNA, lipids and proteins [3,14]. To protect or prevent protection against excess ROS enzymes such as the superoxide dismutase play a crucial role by converting O_2_^•−^ to H_2_O_2_ [3]. Interestingly, population studies provided evidence that OS is correlated with shorter average telomere lengths in white blood cells [16]. However, what about the oxidative stress (OS) impact on women’s oocytes? Starting from the discovery of telomeres until today, different pathways have been suggested to elucidate how OS provokes telomeric DNA damage and shortening. One explanation is that OS induces cell death and/or senescence, and in consequence, in the surrounding vital cells accelerated cell divisions take place as a compensatory mechanism that leads to telomere shortening. However, this process does not reflect the characteristic of telomere shortening and dysfunction in oocytes, since after birth oocytes are non-dividing cells [17,18], and after birth the telomerase activity is largely repressed in somatic tissues [19]. Noteworthy, the telomere length differs across somatic tissues in relation to replicative activity and the quantity of cell divisions is limited upon the “Hayflick limit” at which telomeres become so short leading to cellular senescence [19]. According to another very commonly discussed explanation, ROS triggers (locally at telomeres) single-strand breaks (SSB) [20] or even provokes the collapse of the replication fork and telomere loss in somatic cells [13]. According to Barnes et al., it appears to be possible that oxidative lesions impact the transcription of TERRA at telomeres or the shelterin binding [13]. Research provided evidence that ROS can provoke different types of telomeric DNA damage [20,21], however, due to the high guanine content of the tandem re-peated hexamers (TTAGGG), which is susceptible for oxidation, very often 8-oxoguanine (8-oxoG) is produced (Figure 3) [22,23]. Moreover, those telomeric hexamers are susceptible sites for iron binding too, and iron-mediated biochemical reactions have been suggested to produce hydroxyl radicals which lead to the cleavage at 5′of GGG [24]. In addition to the formation of 8-oxoG, research provided evidence that ROS is able to react with free nucleotide pools in quiescent cells, and interestingly, so called oxidized deoxynucleoside triphosphates (dNTP) seem to be crucial for cell viability and chromosome stability [25]. However, it remains not fully understood yet, how oxidized dNTPs influence telomere maintenance and integrity due to the pleiotropic effect of ROS, and how efficiently oxidative telomeric DNA damage is repaired [26]. Recently it has been reported that a chronic appearance of 8-oxoG at telomeres induces telomere shortening [26], although it was shown by Abey et al., that the enzyme peroxiredoxin1 is highly abundant at telomeres during S phase and is able to protect telomeres from oxidative damage [27].

## 4. Impact of Telomere- and Telomerase-Related Diseases on Oocytes

So far, only few telomere-related diseases have been discovered and have been linked to mutations of some specific genes, such as in DKC1, in TERC and TERT, in NOP10 and NHP2 and in the TINF2 gene [28]. In addition to aplastic anemia and idiopathic lung fibrosis, dyskeratosis congenita is commonly named as a human telomeropathy which is a disease based on mutations in the telomerase [29]. In patients with the latter mentioned disease decreased blood levels of the Anti-Müllerian Hormone was observed what reflects a diminished ovarian reserve [30]. The same was shown in another case report about a woman suffering from dyskeratosis congenita, and additionally, this patient poorly responded to the ovarian stimulation, and telomeres were severely shortened in oocytes and generated embryos [29]. Importantly though, this shows that critical short telomeres upon a disease negatively affect women’s fertility regardless of maternal ageing. In consequence, it appears to be plausible, that other individual factors such as obesity, malnutrition and environmental factors (e.g., smoking) which potentially could influence telomeres or lead to malfunction/mutations of telomerase, could be reflected by a diminished oocyte quality and developmental competence, too.

## 5. Conclusions and Final Remarks

In this Commentary, the current understanding of the impact of oxidative stress on telomere dysfunction and shortening in oocytes of advanced maternal age has been presented. Herein, potential explanations were provided on how elevated ROS alter telomere length homeostasis and biology, and there is no doubt that in human oocytes oxidative telomeric DNA damage appears to be the most relevant cause. However, some questions still remain unelucidated, and future studies on oocytes could aim to reveal if changes in telomere length and function upon ROS are induced directly or indirectly. This novel knowledge would be valuable for developing new therapeutic or prophylactic strategies that protect telomeres from oxidative stress or at least minimize the effects of ROS on telomeres in oocytes of advanced maternal age.
